# The intracellular domain of major histocompatibility class-I proteins is essential for maintaining excitatory spine density and synaptic ultrastructure in the brain

**DOI:** 10.1038/s41598-023-30054-8

**Published:** 2023-04-20

**Authors:** Maciej J. Lazarczyk, Brett A. Eyford, Merina Varghese, Hitesh Arora, Lonna Munro, Tahia Warda, Cheryl G. Pfeifer, Allison Sowa, Daniel R. Dickstein, Timothy Rumbell, Wilfred A. Jefferies, Dara L. Dickstein

**Affiliations:** 1 Nash Family Department of Neuroscience, Icahn School of Medicine at Mount Sinai, New York, NY 10029 USA; 2 Friedman Brain Institute, Icahn School of Medicine at Mount Sinai, New York, NY 10029 USA; 3 Department of Medical Direction and Quality, Division of Institutional Measures, University Hospitals of Geneva, Geneva, Switzerland; 4 Michael Smith Laboratories, The University of British Columbia, 2185 East Mall, Vancouver, BC V6T 1Z4 Canada; 5 The Vancouver Prostate Centre, Robert H.N. Ho Research Centre, Vancouver General Hospital, 2660 Oak Street, Vancouver, BC V6H 3Z6 Canada; 6 Department of Microbiology and Immunology, University of British Columbia, 1365 - 2350 Health Sciences Mall, Vancouver, BC V6T 1Z3 Canada; 7 Centre for Blood Research, University of British Columbia, 2350 Health Sciences Mall, Vancouver, BC V6T 1Z3 Canada; 8 Department of Zoology, University of British Columbia, 2370 - 6270 University Blvd., Vancouver, BC V6T 1Z4 Canada; 9 Department of Medical Genetics, Life Sciences Institute, 1364 - 2350 Health Sciences Mall, Vancouver, BC V6T 1Z3 Canada; 10 Djavad Mowafaghian Centre for Brain Health, University of British Columbia, 2215 Wesbrook Mall, Vancouver, BC Canada; 11 Department of Pathology, Uniformed Services University of the Health Sciences, 4301 Jones Bridge Rd, Bethesda, MD 20814 USA; 12 The Henry M. Jackson Foundation for the Advancement of Military Medicine Inc., Bethesda, MD 20817 USA

**Keywords:** Immunology, Adaptive immunity, Antigen processing and presentation

## Abstract

Major histocompatibility complex class I (MHC-I) proteins are expressed in neurons, where they regulate synaptic plasticity. However, the mechanisms by which MHC-I functions in the CNS remains unknown. Here we describe the first structural analysis of a MHC-I protein, to resolve underlying mechanisms that explains its function in the brain. We demonstrate that Y321F mutation of the conserved cytoplasmic tyrosine-based endocytosis motif YXXΦ in MHC-I affects spine density and synaptic structure without affecting neuronal complexity in the hippocampus, a region of the brain intimately involved in learning and memory. Furthermore, the impact of the Y321F substitution phenocopies MHC-I knock-out (null) animals, demonstrating that reverse, outside-in signalling events sensing the external environment is the major mechanism that conveys this information to the neuron and this has a previously undescribed yet essential role in the regulation of synaptic plasticity.

## Introduction

Although the central nervous system (CNS) has long been considered an “immunologically privileged” organ, MHC-I proteins are expressed in neurons under normal physiological conditions^1–4^. In the brain, MHC-I has been found to play a role in synaptic plasticity, notably in dendrite morphogenesis and synapse pruning^2,5^. Indeed, in the absence of stable surface MHC-I molecules, synapse density is significantly increased^2,6–9^, and memory and learning is impaired^10–13^. These observations interweave the immune system with the neurological system in a yet undefined mechanism.

The cytoplasmic tail of MHC-I has many conserved amino acids that are vital for its function^14,15^. One example is the highly conserved tyrosine-based motif, YXXΦ (where Φ represents any bulky, hydrophobic amino acid). This motif is thought to function as an endocytic sorting signal^14,15^, a basolateral sorting signal and a modulator of MHC-I recycling to the cell surface via tyrosine phosphorylation^16^. A conserved alanine residue at the third position after the tyrosine results in the YXXA motif in MHC-I that we have previously demonstrated is required for recycling MHC-I molecules via tyrosine phosphorylation^17,18^. In leukocyte dendritic cells, point mutation of the tyrosine residue to a phenylalanine residue (Y321F, known hereafter as ΔY) abrogated recycling of MHC-I molecules to endolysosomal compartments, where loading of exogenous peptides occurs. This inability to load peptide impairs cross-presentation of exogenous peptide and prevents the activation of the cytotoxic T-cell response^17^, but the transport of MHC-I to the cell surface and overall expression is normal^17,19,20^. Whether the YXXA motif is relevant for the MHC-I function in neuroplasticity remains unknown.

To discover the role of the MHC-I cytoplasmic tail, and the YXXΦ motif, in the CNS and in particular at the synapse, we examined neuronal morphology and spine and synapse density and morphology in hippocampal neurons from a mouse strain expressing the ΔY mutation. Our structural analysis resolves the debate on the underlying mechanism and supports the conclusion that synaptic plasticity is facilitated by MHC-I in the CNS functioning in *cis* to mediate outside-in function via its intracellular domain.

## Results

### Mutation of the cytoplasmic YXXΦ motif does not affect laminar cell layers

**Figure 1:**
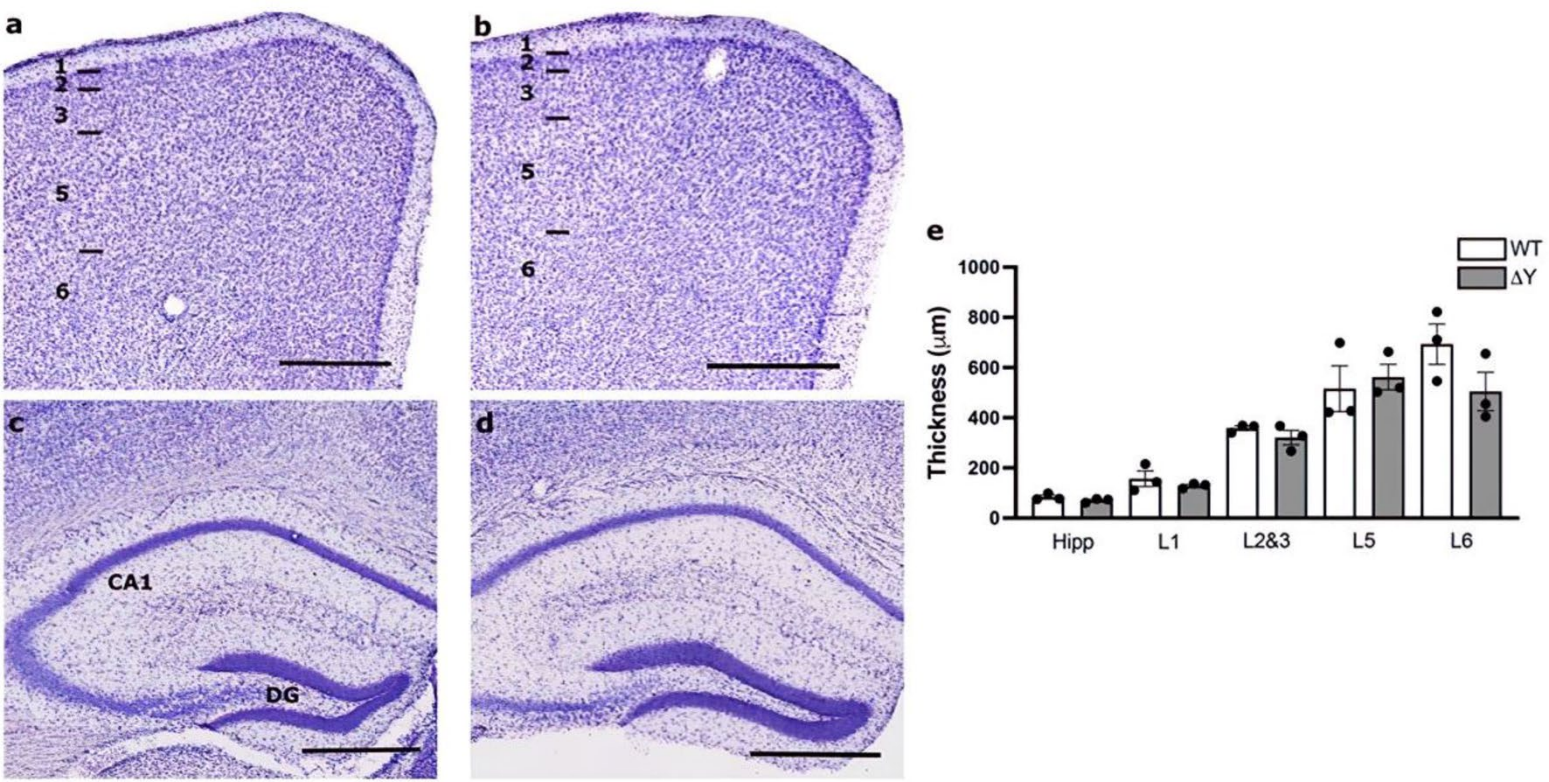
ΔY mutation in MHC-I has no effect on cytoarchitectural stratification. Nissl-stained coronal Sections (50 µm) of adult brains from WT (**a**, **c**) and ΔY (**b**, **d**) mice at the prefrontal and hippocampal levels along the rostrocaudal axis of the neocortex. Layers of the neocortex are represented in Panel a^1–3,5,6^. (**e**) Quantification of layer thickness reveals no differences between WT and ΔY mice. Abbreviations: CA1, *cornu ammonis* 1; DG, dentate gyrus; Hipp, hippocampus; L, layer. Scale bar = 500 µm.

To investigate the effect of the ΔY mutation on brain architecture and lamination, Nissl staining was performed and gross brain morphology was assessed. We found no gross alterations in cortical lamination in ΔY mice. The organization of cortical neurons appeared normal when compared to WT from our previous study^9^, with ΔY mice displaying a process-rich layer I at the pial surface followed by appropriately organized neuronal layers II to VI. (Fig. 1a,b). The cytoarchitecture in the hippocampi of ΔY mice also appeared normal (Fig. 1c,d). Quantification of the thickness of cortical layers in the prelimbic and infralimbic cortex and the pyramidal cell layer of the CA1 regions of the hippocampus showed no differences between genotypes (Fig. 1e). Therefore, the ΔY does not appear to affect neuronal migration.

### Mutation of the cytoplasmic YXXΦ motif does not alter neuronal dendritic morphology

**Figure 2:**
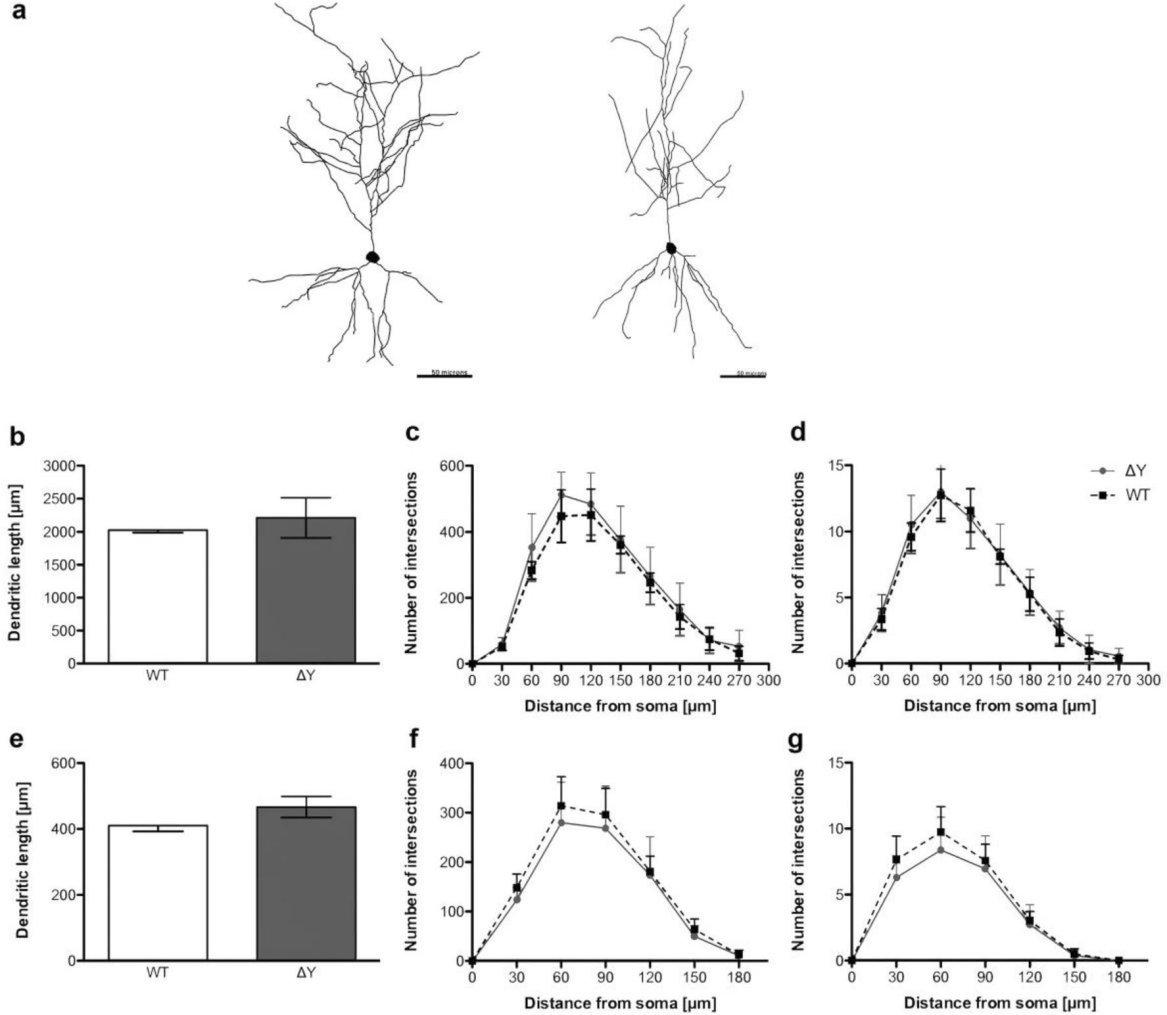
ΔY mutation in MHC-I does not alter neuronal dendritic length and complexity. (**a**) Representative neuron tracings of Lucifer Yellow filled CA1 pyramidal neurons from WT (left) and ΔY mice (right). Scale bar = 50 μm. (**b**) Analysis of apical dendritic length. (**c**, **d**) Sholl analyses of apical dendrites. (**e**) Analysis of basal dendritic length. (**f**, **g**) Sholl analyses of basal dendrites.

We have shown previously that a lack of MHC-I results in a decrease in the length and complexity of apical dendrites of CA1 pyramidal neurons upon aging^9^. To test whether the ΔY mutation would have a similar effect as the absence of MHC-I, we quantified dendritic length and complexity in ΔY and WT mice (Fig. 2a). A total of 11 animals and approximately 5 neurons per animal met the inclusion criteria for use in neuronal reconstructions. In contrast with our previous findings in MHC-I knock-out or null mice (K^b^D^b−/−^) where we reported significantly shorter apical dendrites and decreased neuronal complexity compared to WT^9^, our results showed that the ΔY mutation has no effect on dendritic length nor on dendritic complexity as indicated from a Sholl analysis on dendritic length and number of intersections in both apical and basal dendrites (Fig. 2b–g).

### Mutation of the cytoplasmic YXXΦ motif alters density of dendritic spines and synapses

**Figure 3:**
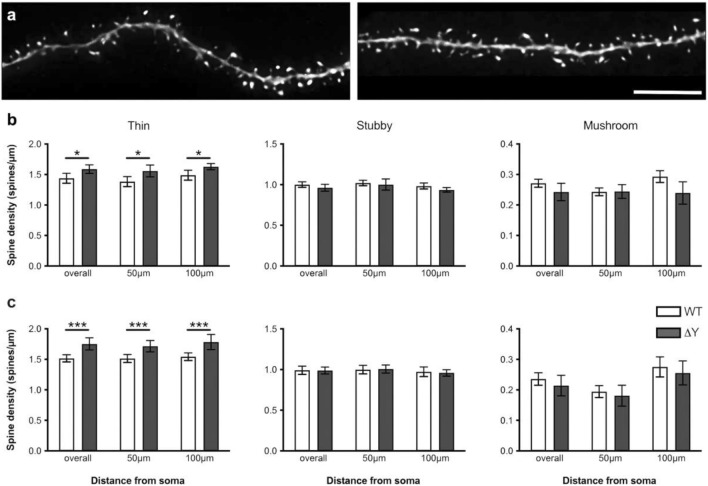
ΔY mutation in MHC-I increases the density of thin spines (**a**) Representative confocal images of apical dendritic segments of CA1 pyramidal neurons from WT (left) and ΔY mice (right). Scale bar = 5 μm. There was a significant increase in the density of (**b**) apical and (**c**) basal thin spines in ΔY compared to WT mice. There was no change in the density of stubby and mushroom spines. Note the different scales on the Y-axis for each spine type. *P < 0.05, ***P < 0.001. Data represent means ± SEM.

Dendritic spine complexity as well as spine morphology influences synaptic function, and changes in spine size can play a substantial role in mediating cognitive function^21^. Our previous data in K^b^D^b-/-^ mice found that MHC deficiency significantly increases the density of thin spines and significantly decreases the density of stubby spines in apical dendrites^9^. Therefore, we sought to determine whether the ΔY mutation affects dendritic spines in the same manner. We analyzed a total of 19,846 spines (9,752 apical and 10,094 basal) from ΔY mice and 23,328 spines (11,673 apical and 11,645 basal) from WT mice (3 dendritic segments/apical or basal dendrite/neuron, n = 6 neurons for WT and n = 5 neurons for ΔY) (see Fig. 3a for representative images). Our analysis revealed significant increases in the density of apical thin spines (t_(4)_ = 4.289, *p* = 0.0128) in ΔY mice compared to WT mice (Fig. 3b). There were no differences in the density of stubby or mushroom spines (Fig. 3b). Analysis of basal spines showed similar results, with significant increases in the density of basal thin spines (t_(4)_ = 10.39, *p* < 0.0005) in ΔY mice compared to WT mice and no changes in both stubby and mushroom spine density (Fig. 3c).

**Figure 4:**
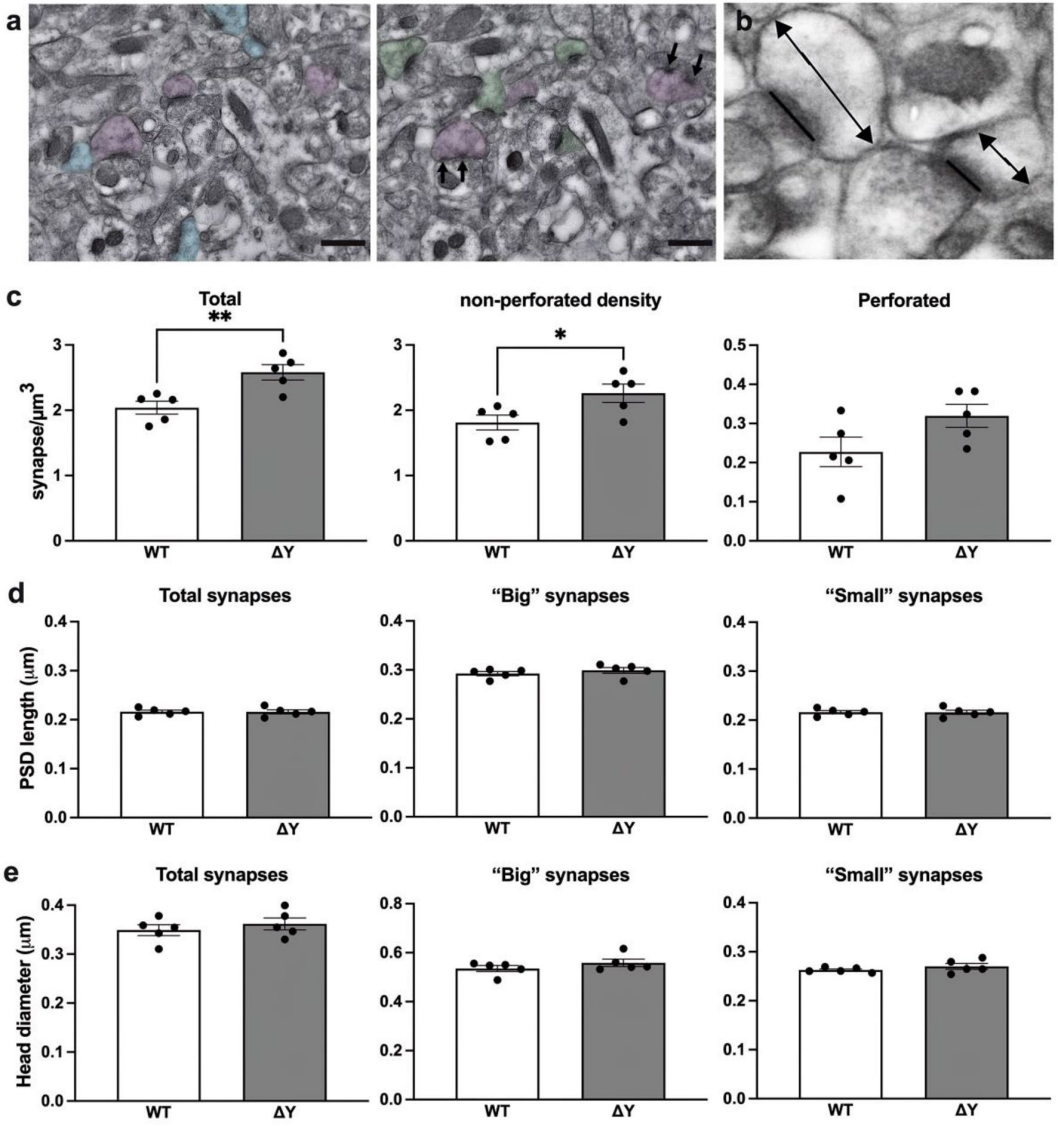
ΔY* MHC-I* mutation result in an increase in synapse density but does not affect PSD length or spine HD. (**a**) Electron micrographs depicting synapses counted using the disector method. Only asymmetric axospinous synapses that were present in the reference panel (green or blue), but not in the look-up panel were counted. Synapses present in both panels (purple) are not included. Non-perforated synapses are distinguished from perforated synapses by the presence of a discontinuity within the PSD (arrows). Scale bars = 500 nm. (**b**) EM micrograph illustrating how PSD length (straight line) and HD (line with arrows) was measured. (**c**) ΔY mice exhibited an increase in total synapse and non-perforated synapse density compared to WT. No differences were observed in perforated synapse density. No differences were observed in (**d**) PSD length or (**e**) HD of total, “small” and “big” synapses in ΔY mice compared to WT. *P < 0.05. **P < 0.01. Data represent means ± SEM.

Having found that disruption of YXXΦ motif affects the density of thin spines, we subsequently examined its impact on spine morphology at the ultrastructural level using quantitative electron microscopy. Synapse density, spine head diameter (HD), and postsynaptic density (PSD) length in the *stratum radiatum* layer of the CA1 were determined using a stereologically unbiased disector method (Fig. 4a,b). In total 4712 synapses were counted (2633 for ΔY group, approximately 527/animal; 2079 for WT group, approximately 416/animal), in order to estimate synapse density, and 2079 synapses were measured (1147 for ΔY group, approximately 229/animal; 932 for WT group, approximately 186/animal), in order to determine spine HD and PSD length. A *t*-test revealed a significant increase in total synapse density in ΔY mice compared to WT (2.58 ± 0.11, and 2.03 ± 0.09 synapse/μm^3^, respectively, t_(8)_ = 3.542, *p* = 0.008; Fig. 4c). The extent of this increase was similar to that observed in our previous studies comparing K^b^D^b−/−^ mice to WT^9^. Further analysis performed to identify non-perforated and perforated synapses revealed that the observed increase in density occurred only in non-perforated synapses (2.26 ± 0.14, and 1.81 ± 0.11 synapse/μm^3^, respectively; *t*_(8)_ = 2.496, *p* = 0.037) and not in perforated synapses (0.31 ± 0.03, and 0.22 ± 0.04 synapse/μm^3^, respectively; *t*_(8)_ = 1.935, *p* = 0.089; Fig. 4c). Thereafter, morphometric analysis of the postsynaptic compartment was performed, and the maximum PSD length and spine HD were measured in all synapses*.* As we have previously shown, the effect of MHC-I-deficiency on synapses depends on their type, with mushroom spines, possessing larger and/or perforated synapses, remaining relatively spared^9^. Therefore, in order to analyze the significance of MHC-I cytoplasmic domain for maintenance of the morphology of postsynaptic compartment, we analyzed “small” and “big” synapses separately. Using an unbiased k-means clustering method, the spines forming synapses were classified into one of the two categories, small or big, depending on their maximum HD (the threshold value: 387.5 nm). As far as the cluster of big synapses was concerned, analysis of maximum PSD length revealed no differences between the genotypes (ΔY = 0.299 ± 0.006 μm *versus* WT = 0.292 ± 0.004 μm, *p* = 0.39; Fig. 4d). Similarly, there was no difference concerning the maximum spine HD in this cluster (ΔY = 0.558 ± 0.015 μm versus WT = 0.535 ± 0.012 μm, *p* = 0.26; Fig. 4e). In regards to maximum PSD length and spine head diameter in the cluster of small synapses, MHC-I Y321F substitution, although sufficient to increase synapse density (Fig. 4c), had no impact on maximum PSD length and spine head diameter in the cluster of small synapses (PSD length: ΔY = 0.178 ± 0.007 μm versus WT = 0.182 ± 0.003 μm, *p* = 0.42; spine head diameter: ΔY = 0.270 ± 0.013 μm versus WT = 0.263 ± 0.002 μm, *p* = 0.27; Fig. 4d,e).

## Discussion

In this study, we have discovered the a mechanism underlying synaptic integrity and linked it to a single tyrosine in the cytoplasmic tail of MHC-I proteins. We have demonstrated previously that the absence of MHC-I resulted in significant reduction in dendritic complexity and changes in synaptic density. However, the mechanism by which this occurs and the implication of distinct MHC-I domains in this effect remained unknown. It is possible that MHC-I may elicit its role in synaptic plasticity via the cytoplasmic domain. The role of this domain in neurons is poorly understood. In non-neuronal cells, the cytoplasmic domain of MHCI is involved in the regulation of toll-like receptor (TLR) signaling, and cross-presentation of exogenous peptides^17,22^. In the present study, we found that the cytoplasmic domain, and notably the ΔY residue, is crucial to specific neuronal functions of MHC-I in the hippocampus. Our studies with mice expressing MHC-I that have a single amino acid mutation in a tyrosine within the cytoplasmic tail phenocopies and mirrors the synaptic changes that occur with the MHC-I knock-out or null mutation, supporting the conclusion that this structure within the MHC-I molecule is responsible for mediating synaptic plasticity by outside-in or reverse signalling via its intracellular domain. This resolves the debate in the field regarding the mechanism acting in *cis* or exclusively in *trans*.

Our experiments demonstrate that ΔY MHC-I mice have increased thin spines and synapse density in the CA1 region of the hippocampus, although they do not show changes in complexity of neurons, dendritic length or synaptic ultrastructure, such as PSD length and spine head diameter, when compared to WT mice. Taken together with our previous observations in K^b^D^b−/−^ mice^9^, these results indicate that the Y321 residue in the cytoplasmic domain may be crucial for MHC-I effect on synaptic pruning since there was an increase in the amount of thin spines. it has been established that there is an age-related loss of thin spines with aging in animal models such as non-human primates^23,24^ and a maintenance or loss with age in the CA1 or some rodent models of aging ^25,26^. Interestingly, as the Y321F mutation does not affect cell surface MHC-I expression^17^, it suggests that the extracellular MHC-I domain is not sufficient, and therefore, might be not implicated in the synapse pruning. However, the cytoplasmic domain appears not to be involved in the mediation of the role of MHC-I in dendrite and synapse morphology. Indeed, we demonstrate here that whereas the absence of MHC-I results in decreased total length and complexity of apical dendrites, fewer stubby spines, and reduced PSD length and head diameter^9,27^, a disruption of the cytoplasmic domain of MHC-I has no such effect.

The tyrosine mutation in MHC-I does not affect cell surface expression; however, the possibility exists that the extracellular MHC-I domain is not involved in the synapse pruning. In a more complex interpretation of our results, the mutating the intracellular tyrosine residue could cause surface MHC-I to impair protein–protein interaction with scaffolds that in turn, effect the ratio of synaptic compared to the extra-synaptic localization and thereby, indirectly prevent the extracellular domain of MHC-I to function normally. It is presently unclear whether this takes place, but this point is worth experimentally addressing in the future.

In summary, here we report that mechanistically the highly conserved tyrosine in the cytoplasmic tail of MHC-I acts in outside-in or reverse signaling to mediate synaptic maintenance and function, including synapse and spine densities. These findings directly link mechanisms underpinning synaptic plasticity precisely to the single amino acid level. Overall, these findings reinforce the importance of MHC-I expression in the CNS in maintaining synaptic structure and thereby plasticity upon aging and corroborate the dual role of MHC-I molecules in both the immune system and in the CNS. Finally, this work notably validate MHC-I modulators of MHC-I expression or of MHC-I signalling via the cytoplasmic tail to investigate new modalities to maintain spine morphology and synapse density and thereby modify synaptic function in learning and memory that may be translated clinically.

## Materials and methods

### Experimental animals

The study has been reported in accordance with ARRIVE guidelines^28^ and all mouse experiments were approved by the Animal Care Committee at UBC. C3H/He mice expressing transgenic wild type (WT) MHC-I allele H-2 K^b^ (WTK^b^C3H) or cytoplasmic tail tyrosine mutant (ΔYK^b^C3H) have been previously described^17^. The ΔYK^b^ H2D^b−/−^ and WTK^b^ H2D^−/−^ strains were generated by crossing H2K^b^2D^b^ double knockout (H2K^-/-^D^-/-^) with WTK^b^C3H and ΔYK^b^C3H strains, respectively. WTK^b^C3H and ΔYK^b^C3H strains were originally maintained on a C3H/He background and express MHC-I genes H2K^k^ and H2D^k^ as well as the knocked in transgenic H-2 K^b^ gene (ΔYK^b^ or WTK^b^). The mice were backcrossed onto a H2K^-/-^D^-/-^ background (H2K^b^2D^b^ double knockout) for at least 10 generations while the original line was retired. Progeny of the crosses were genotyped by PCR and phenotyped by flow cytometry to establish the presence of MHC-I alleles H2K^k^, K^b^, D^k^ and D^b^ as well as the disrupted H-2 K^b^ and D^b^ genes. PCR to detect presence of knocked in transgenic H-2 K^b^ (ΔYK^b^ or WTK^b^) used H-2 K^b^ specific oligonucleotides, 5′-TCGCTGAGGTATTTCGTC-3′ and 5′-TTGCCCTTGGCTTTCTG T-3′. Primers used to detect MHC-I alleles H-2K^k^ and D^k^ were H2K&D^k^_fwd: 5'- GGAAGCCCCGGTTCATCTCT -3′, H2K^k^_rev: 5′-ACAGCCGTACATCCGTTGGAAC-3' and H2D^k^_rev: 5′-CCGGACAACCGCTGG ATC-3′^29–31^. Expression of specific MHC-I molecules on the cell surface of lymphocytes was assessed by flow cytometry. Blood was collected from the saphenous vein in heparin-coated microvette tubes. To isolate peripheral blood leukocytes (PBLs), whole blood was transferred with 200 μL of phosphate-buffered saline (PBS) to BD Falcon 5 mL polystyrene round-bottom tubes. An 800 μL Ficoll gradient was applied and tubes were spun for 15 minutes at 500 ×  *g*. PBLs were recovered and washed twice in RPMI complete media (RPMI-1640 supplemented with 10% fetal calf serum). PBLs from progeny of H2K^b^2D^b^ double knockout (H2K^−/−^D^−/−)^ with WTK^b^C3H and ΔYK^b^C3H strains were labelled using antibodies specific to extracellular domains of H-2 K^b^, K^k^ and D^k^, respectively, and phenotyped by flow cytometry to establish the presence of MHC-I alleles H-2K^k^, K^b^, D^k^ and D^b^ as well as the disrupted H-2 K^b^ and D^b^ genes.

Twelve-month-old mice were used for all neuronal and synapse morphology experiments. As our previous study showed no differences between males and females, mice of both sexes were included in this study. Mice were kept under a 12-h light/dark cycle and fed standard lab chow and water ad libitum. All animal experiments and methods were performed at the University of British Columbia, Vancouver, BC, Canada and were conducted in compliance with the University of British Columbia Animal Care Committee under the direction of the Canadian Council for Animal Care.

### Perfusion and tissue processing

Mice were anesthetized with a mixture of Ketamine (100 mg/kg) and Xylazine (10 mg/kg) administered intraperitoneally, and transcardially perfused with 1% paraformaldehyde in PBS, followed by 4% paraformaldehyde with 0.125% glutaraldehyde in PBS as described previously^9,32,33^. The brains were carefully dissected, postfixed overnight at 4 °C in 4% paraformaldehyde in PBS with 0.125% glutaraldehyde and cut on a Vibratome (Leica, Buffalo Grove, IL). Brains samples were cut into 200 μm thick sections for neuronal morphology and spine analysis, 250 μm thick sections for electron microscopy (EM) and synapse analysis, and 50 μm sections for cytoarchitecture assessment. All sections were stored at 4 °C in PBS with 0.01% sodium azide until ready for use.

### Cytoarchitecture assessment

Neuronal cells in the neocortex and hippocampus were observed by Nissl staining to assess cytoarchitecture of cortical layers. Briefly, 50 μm thick sections were stained in 0.5% cresyl violet in 0.3% acetic acid, washed in distilled water, dehydrated in graded concentrations of ethanol (70%, 80%, 90% and 100%), cleared in xylene and coverslipped. Images were taken on a Zeiss Axiophot 1 microscope equipped with a motorized stage, video camera system, and StereoInvestigator software (v. 2020.1, MBF Bioscience, Williston, VT). Cortical layer thickness was measured for each layer of prefrontal sections encompassing the prelimbic and infralimbic cortex, and the CA1 pyramidal layer of the hippocampus using StereoInvestigator software (MBF Biosciences). A contour was drawn around each layer, according to the cytoarchitecture as described in the Paxinos and Franklin mouse atlas (ref)mmm m. Using the measure line tool, a line was placed perpendicular to the pial-facing edge of contour and the thickness was then measured from the top to bottom of each layer at multiple, equidistantly spaced locations throughout the entirety of the contour (8 to 21 sites, depending on layer size). Layers were measured in up to four sections per individual and averaged.

### Intracellular dye injections

For intracellular injections, sections were incubated in 4′,6-diamidino-2-phenylindole (DAPI, Sigma, St. Loius, MO) for 5 min to reveal the cytoarchitectural features of the pyramidal layer of CA1 of the hippocampus. The sections were then mounted on nitrocellulose membranes and immersed in PBS. Neurons were impaled with a sharp glass micropipette and loaded with 5% Lucifer Yellow in distilled water under a direct current of between 3 and 8 nA for 5–10 min, or until dye had completely filled distal processes and no further loading was observed, as described previously^9,32,33^. Five to ten neurons were loaded per animal and spaced far enough apart from each other to prevent overlapping of dendrites. The sections were then mounted on gelatin-coated glass slides and cover slipped in Fluoromount G (Southern Biotech, Birmingham, AL).

### Neuron and dendritic reconstruction

To be included in the analysis, a loaded neuron had to satisfy the following criteria: (1) reside within the pyramidal layer of the CA1 as defined by cytoarchitectural characteristics; (2) demonstrate complete filling of dendritic tree, as evidenced by well-defined endings; and (3) demonstrate intact tertiary branches, with the exception of branches that extended beyond 50 μm in radial distance from the cell soma^9,32,33^. Neurons meeting these criteria were reconstructed in three-dimensions (3D) with a 40 × /1.4 N.A., Plan-Apochromat oil immersion objective on a Zeiss Axiophot 2 microscope equipped with a motorized stage, video camera system, and Neurolucida morphometry software (MBF Bioscience, Williston, VT). Using Neurolucida Explorer software (MBF Bioscience) total dendritic length, number of intersections, and the length of dendritic material per radial distance from the soma, in 30 μm increments were analyzed in order to assess neuronal morphological diversity and potential differences among animals^34^. Personnel undertaking the reconstructions were blinded to the group genotypes.

### Confocal laser scanning microscopy and spine acquisition

Using an approach that precludes sampling bias of spines, dendritic segments were selected with a systematic-random design^9,32,33^. Investigators who imaged dendritic segments and analyzed spines were blinded to the group genotypes. Dendritic segments, 20–25 μm in length, on secondary and higher order branches and at 50 and 100 μm from the soma, were imaged on the Zeiss LSM 510 confocal microscope (Zeiss, Thornwood, NY) using a 100x/1.4 N.A. Plan-Apochromat objective with a digital zoom of 3.5 and an Ar/Kr laser at an excitation wavelength of 488 nm. All confocal stacks were acquired at 512 × 512 pixel resolution with a z-step of 0.1 μm and approximately 1 μm above and below the identified dendritic segment, a pinhole setting of 1 Airy unit and optimal settings for gain and offset. On average 3 z-stacks were imaged per apical and basal tree and 5 neurons per animal. In order for a dendritic segment to be optimally imaged it had to satisfy the following criteria: (1) the entire segment had to fall within a depth of 50 μm; (2) dendritic segments had to be either parallel or at acute angles to the coronal surface of the section; and (3) segments did not overlap other segments that would obscure visualization of spines^9,32,33^. To improve voxel resolution and reduce optical aberration along the Z-axis, the acquired images were deconvolved using an interactive blind deconvolution algorithm (AutoDeblur version 8.0.2; MediaCybernetics, Rockville MD).

### Spine analysis

After deconvolution, the confocal stacks were analyzed using NeuronStudio software^35,36^ (http://www.mssm.edu/cnic) to examine global and local morphometric characteristics of dendritic spines, such as density, shape (stubby, mushroom, and thin), diameter and head volume. This software allows for automated digitization and reconstructions of 3D neuronal morphology from multiple confocal stacks on a spatial scale and averts the subjective errors encountered during manual tracing using a Rayburst-based spine analysis^35,36^. A spine was labelled thin or mushroom if the ratio of its maximum head diameter to maximum neck diameter was > 1.1. Spines that fit those criteria and had a maximum head diameter of < 0.35 µm were classified as thin spines, and otherwise they were classified as mushroom spines.

### Electron microscopy

Coronal sections encompassing the CA1 region of the hippocampus were prepared for EM as reported previously^9,33,37^. Briefly, slices were cryoprotected in graded PBS/glycerol washes at 4 °C, and manually microdissected to obtain blocks containing the CA1 region. The blocks were rapidly freeze-plunged into liquid propane cooled by liquid nitrogen (− 190 °C) in a universal cryofixation system KF80 (Reichert-Jung, Depew, NY) and subsequently immersed in 1.5% uranyl acetate dissolved in anhydrous methanol at − 90 °C for 24 h in a cryosubstitution unit (Leica). Block temperatures were raised from − 90 to − 45 °C in steps of 4 °C/hour, washed with anhydrous methanol, and infiltrated with Lowicryl resin (Electron Microscopy Sciences, Hatfield, PA) at − 45 °C. The resin was polymerized by exposure to ultraviolet light (360 nm) for 48 h at − 45 °C followed by 24 h at 0 °C. Block faces were trimmed and ultrathin Sects. (90 nm) were cut with a diamond knife (Diatome, Hatfield, PA) on an ultramicrotome (Reichert-Jung) and serial sections were collected on formvar/carbon-coated nickel slot grids (Electron Microscopy Sciences).

### Quantitative analyses of synapse density

For synapse quantification, serial section micrographs were imaged at 15000 × on a Hitachi H-7000 transmission electron microscope using an AMT Advantage CCD camera (Advanced Microscopy Techniques, Danvers, MA). Nine sets of serial images across the same set of 5 consecutive ultrathin sections were taken from the *stratum radiatum* of the hippocampal CA1 field and imported into Adobe Photoshop (version CS5, Adobe Systems, San Jose, CA). To obtain a stereologically unbiased population of synapses for quantitative morphologic analysis, we used a disector approach on ultrathin sections as in previous reports^9,33,38^. Briefly, all axospinous synapses were identified within the first and last 2 images of each 5-section serial set, and counted if they were contained in the reference image but not in the corresponding look-up image. To increase sampling efficiency, the reference image and look-up image were then reversed; thus each animal included in the current study contributed synapse density data from a total of 18 disector pairs. The total volume examined was 11.317 μm^3^, and the height of the disector was 180 nm. Axospinous synapse density (per μm^3^) was calculated as the total number of counted synapses from both images divided by the total volume of the disector. The criteria for inclusion as an axospinous synapse included the presence of synaptic vesicles in the presynaptic terminal and a distinct asymmetric PSD separated by a clear synaptic cleft. The synapse density was calculated as the total number of counted synapses divided by the total volume of the disectors used. For a synapse to be scored as perforated, it had to display two or more separate PSD plates. Other ultrastructural synaptic parameters including PSD length and maximum spine HD were determined using a method previously described^33,38^. Briefly, all axospinous synapses in the middle portion of three serial sections were identified. Then, for each synapse, the longest PSD length and spine HD in 3 serial sections was identified and measured. For perforated synapses, the lengths of all PSD segments were summed and the total length was used in the statistical analyses. All electron microscopy imaging and analyses were carried out by the operator blinded to the group genotypes.

### Statistical analysis

For all the morphology studies, mean values from single cells were obtained and then averaged for each animal and were used for comparison of means. Synapse densities, PSD length, and synapse head diameter were analyzed using a one-way ANOVA with Bonferroni’s *post-hoc* tests comparing each genotype to the other. All data were represented as mean ± SEM. All statistical analyses were carried out using Prism software (GraphPad).

## Data Availability

The datasets used and/or analysed during the current study available from the corresponding author on reasonable request.

## Abbreviations

ΔY: Tyrosine 321 to Phenylalanine (Y321F)
β2m: β2-Microglobulin
CA1: *cornu ammonis* 1
CNS: Central nervous system
DC: Dendritic cells
EGFR: Epidermal growth factor receptor
HD: Head diameter
MHC-I: Major histocompatibility complex class I
NMDA: *N*-Methyl-D-aspartate
PBS: Phosphate-buffered saline
PSD: Postsynaptic density
WT: Wild type
